# Nitric oxide and superoxide mediate diesel particle effects in cytokine-treated mice and murine lung epithelial cells — implications for susceptibility to traffic-related air pollution

**DOI:** 10.1186/1743-8977-9-43

**Published:** 2012-11-15

**Authors:** Nicholas D Manzo, Adriana J LaGier, Ralph Slade, Allen D Ledbetter, Judy H Richards, Janice A Dye

**Affiliations:** 1 Department of Molecular Biomedical Sciences, College of Veterinary Medicine, North Carolina State University, Raleigh, NC, 27606, USA; 2 Department of Biological Sciences, College of Arts and Sciences, Florida Gulf Coast University, 10501 FGCU Blvd, Fort Myers, FL, 33965, USA; 3 Cardiopulmonary and Immunotoxicology Branch (CIB), Environmental Public Health Division, National Health and Environmental Effects Research Laboratory, ORD, U.S. Environmental Protection Agency, Research Triangle Park, NC, 27711, USA; 4 Inhalation Toxicology Facilities Branch (ITFB), Environmental Public Health Division, National Health and Environmental Effects Research Laboratory, ORD, U.S. Environmental Protection Agency, Research Triangle Park, NC, 27711, USA; 5 Current address: Division of Pulmonary, Allergy and Critical Care, Duke University Medical Center, 2075 MSRBII, 106 Research Drive, DUMC Box 103000, Durham, NC, 27710, USA

**Keywords:** Traffic, Diesel, Particles, Epithelial cells, Phagocytes, Nitric oxide, Peroxynitrite, Redox balance

## Abstract

**Background:**

Epidemiologic studies associate childhood exposure to traffic-related air pollution with increased respiratory infections and asthmatic and allergic symptoms. The strongest associations between traffic exposure and negative health impacts are observed in individuals with respiratory inflammation. We hypothesized that interactions between nitric oxide (NO), increased during lung inflammatory responses, and reactive oxygen species (ROS), increased as a consequence of traffic exposure ─ played a key role in the increased susceptibility of these at-risk populations to traffic emissions.

**Methods:**

Diesel exhaust particles (DEP) were used as surrogates for traffic particles. Murine lung epithelial (LA-4) cells and BALB/c mice were treated with a cytokine mixture (cytomix: TNFα, IL-1β, and IFNγ) to induce a generic inflammatory state. Cells were exposed to saline or DEP (25 μg/cm^2^) and examined for differential effects on redox balance and cytotoxicity. Likewise, mice undergoing nose-only inhalation exposure to air or DEP (2 mg/m^3^ × 4 h/d × 2 d) were assessed for differential effects on lung inflammation, injury, antioxidant levels, and phagocyte ROS production.

**Results:**

Cytomix treatment significantly increased LA-4 cell NO production though iNOS activation. Cytomix + DEP-exposed cells incurred the greatest intracellular ROS production, with commensurate cytotoxicity, as these cells were unable to maintain redox balance. By contrast, saline + DEP-exposed cells were able to mount effective antioxidant responses. DEP effects were mediated by: (1) increased ROS including superoxide anion (O_2_˙^-^), related to increased xanthine dehydrogenase expression and reduced cytosolic superoxide dismutase activity; and (2) increased peroxynitrite generation related to interaction of O_2_˙^-^ with cytokine-induced NO. Effects were partially *reduced* by superoxide dismutase (SOD) supplementation or by blocking iNOS induction. In mice, cytomix + DEP-exposure resulted in greater ROS production in lung phagocytes. Phagocyte and epithelial effects were, by and large, *prevented* by treatment with FeTMPyP, which accelerates peroxynitrite catalysis.

**Conclusions:**

During inflammation, due to interactions of NO and O_2_˙^-^, DEP-exposure was associated with nitrosative stress in surface epithelial cells and resident lung phagocytes. As these cell types work in concert to provide protection against inhaled pathogens and allergens, dysfunction would predispose to development of respiratory infection and allergy. Results provide a mechanism by which individuals with pre-existing respiratory inflammation are *at increased risk* for exposure to traffic-dominated urban air pollution.

## Background

Exposure to traffic emissions is associated with adverse health outcome [1], including increased respiratory infections and asthmatic and allergic symptoms [2-4]. Associations are based on various traffic exposure metrics including near road black carbon levels [5], fine particulate matter (PM_2.5_) absorbance [6], nitrogen dioxide [7,8] and carbon monoxide [9] concentrations, traffic density [10], residential [6,11,12] or school [13] proximity to major roadways, and combined assessments [14,15].

In urban areas, diesel exhaust particles (DEP) comprise a significant amount of the airborne PM_**2.5**_ associated with traffic emissions [16]. DEP are generated by the incomplete combustion of fossil fuel, and are composed of a carbonaceous core onto which variable amounts of organic carbon (OC)-based compounds [e.g., quinones, polycyclic aromatic hydrocarbons (PAHs)] and roadway-associated metals are adsorbed [16,17]. These DEP subcomponents are capable of producing reactive oxygen species (ROS) either directly or secondarily via effects on cellular production of oxidants [18,19].

The strongest associations between traffic exposure and negative impacts on health are observed in individuals with pre-existing respiratory conditions. Conditions include acute bronchitis [20], chronic bronchitis [7], chronic rhinitis [21], chronic obstructive pulmonary disease (COPD) [20], atopy and allergic sensitization [6,22] and, in particular, asthma [12,14]. In asthmatics, especially children [13], traffic exposure is associated with increases in asthma symptoms [10,11], asthma severity [12], emergency department visits [9,15], hospitalization [8,12], and declines in pulmonary function [14].

Biologic mechanism(s) responsible for the greater health effects observed in these at-risk populations are not fully understood. Increasing evidence suggests that PM effects are, in large part, mediated by excessive reactive oxygen species (ROS) production [23,24]. Relatedly, we exposed lung epithelial cells to an OC-rich DEP sample (as a surrogate for traffic particles) and showed that, on an equi-mass basis, it induced greater cytotoxicity than did simple elemental carbon (EC)-based particles. We further demonstrated that if the epithelial cells were first established within an inflammatory microenvironment, exposure to the OC-rich DEP, but not the EC particles, resulted in overt oxidative stress, leading to significant epithelial damage and solute barrier dysfunction [25].

The question remained, however, as to why comparable exposure to traffic-based particles resulted in greater injury in the *inflamed* cells ─ and by extension ─ why disproportionate respiratory health effects occur in exposed *at-risk* individuals? A common feature across these inflammatory lung disorders is that epithelial cells lining the respiratory tract are continually exposed to mediators from inflammatory cells. This, in turn, results in epithelial cell activation, with subsequent production of secondary mediators [e.g., chemokines, nitric oxide (NO)] [26]. NO is a critical intra- and intercellular messenger. In health, constitutive expression of NO synthases (nNOS and eNOS) by lung epithelial cells and other cell types serve to maintain basal lung NO levels; thereby regulating airway tone and patency [27]. Under inflammatory conditions, however, NO production can be greatly increased (up to 1,000-fold) via activation of inducible NOS (iNOS). As a free radical, NO can be oxidized, reduced, or complexed with other biomolecules ─ with high levels contributing directly to tissue injury [27].

**Figure 1:**
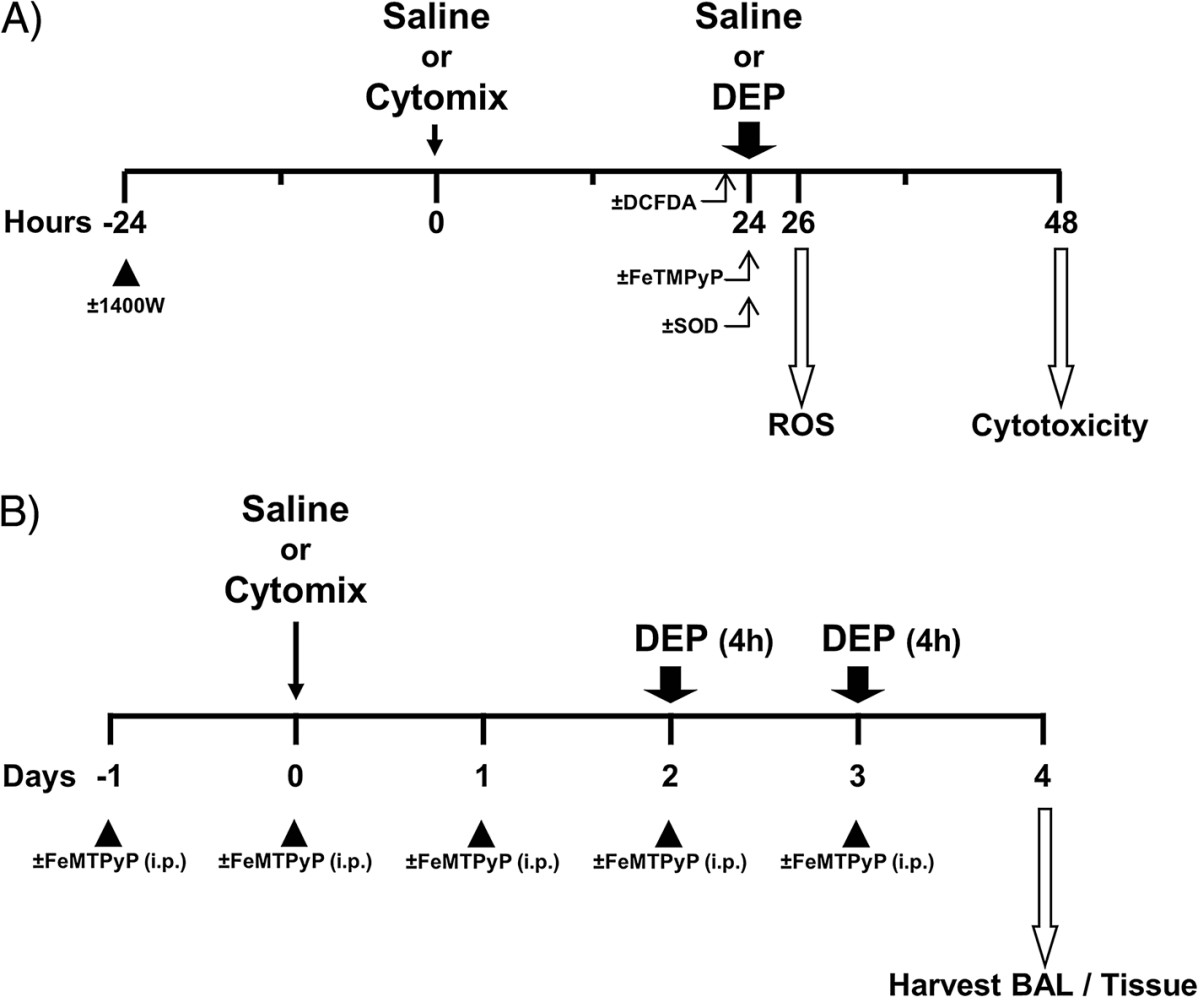
**Exposure time line.** (**A**) Confluent LA-4 epithelial cells treated with cytomix (TNFα + IL-1β + IFNγ) × 24 h followed by DEP (25 μg/cm^2^) for 2 h (fluorescent end points) or 24 h (cytoxicity). (**B**) Exposure time line for BALB/c mice treated via oropharyngeal aspiration with PBS or cytomix (Day 0); exposed to air or DEP (2 mg/m^3^ 4 h/d × 2 d) (Day 2 and 3) and necropsied on Day 4. A subset of mice received FeTMPyP systemically (10 mg/kg, i.p.) (Day −1 to 4).

We hypothesized, therefore, that a key biological mechanism underlying susceptibility of at-risk individuals to traffic-based emissions relates to interactions between (1) particle-associated ROS and (2) endogenous mediators - in particular NO, which is often increased within inflamed airways and deep lung spaces. To test this hypothesis, we again utilized an *in vitro* approach wherein murine alveolar type II-like lung epithelial (LA-4) cells were pretreated with a combination of pro-inflammatory cytokines (TNFα + IL-1β + IFNγ) to create a *generic* inflammatory microenvironment. We have previously demonstrated that LA-4 cells stimulated with this cytokine mixture (referred to as *cytomix*) have increased production of the chemokines MIP-2 and RANTES by ≥ 5-fold [25]. Herein, we further assess the effects of cytomix on LA-4 cell NO production. Then, using OC-rich DEP (again as a surrogate for particle-phase components of traffic emissions), we examine differential effects of DEP exposure on saline- vs. cytomix-treated cells in terms of cytotoxicity and changes in intracellular ROS production, superoxide anion (O_2_˙^-^) production, and cell antioxidant levels. Additionally, because NO can react with O_2_˙^-^ to produce the longer-lived oxidant, peroxynitrite (ONOO^-^) [28], subsets of cells were co-treated with various blockers and agents including FeTMPyP which catalyzes decomposition of ONOO^-^ (Figure 1A).

In a like manner to the *in vitro* studies, BALB/c mice were given a cytokine mixture via oropharyngeal aspiration to establish a *generic* lung inflammatory state. Two days later, at the peak of the lung inflammatory response, saline- or cytokine-treated mice underwent nose-only DEP inhalation exposures for two consecutive days. Twenty four hours later, mice were assessed for differential effects of DEP exposure on (1) lung injury and inflammation and (2) changes in lung antioxidant levels and ROS production in cells obtained via bronchoalveolar lavage (BAL). As above, a subset of mice received systemic FeTMPyP to evaluate whether ONOO^-^ production contributed to DEP-induced effects (Figure 1B).

Our results suggest that traffic-based air pollutant health effects are mediated by a complex interplay between the radical-generating potential of inhaled traffic-source PM which, in concert with mediators from ongoing lung inflammatory processes, cooperate to alter and disrupt antioxidant defenses of lung surface epithelial cells and phagocytic cell populations.

## Results

### Nitric oxide production in cytomix-treated LA-4 cells

**Figure 2:**
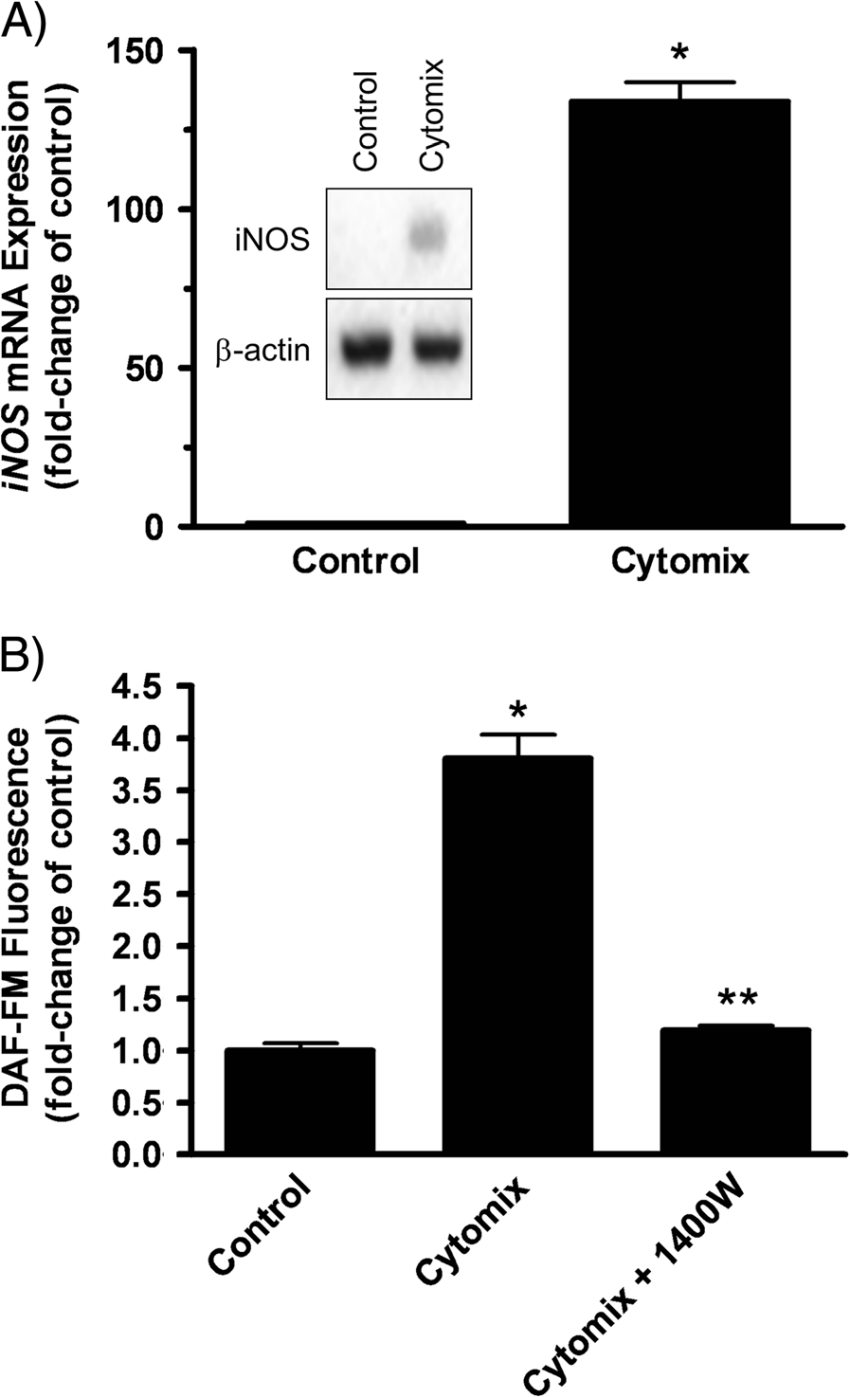
**Cytomix treatment of LA-4 cells increases iNOS and NO production.** (**A**) iNOS mRNA and protein (inset) expression after 24 h post-cytomix treatment. Data are expressed as the mean fold increase (± SEM) over control cells and is representative of three independent experiments. (**B**) Fluorescence of DAF-FM diacetate oxidation by NO, 24 h post-cytomix treatment, in the presence of 1400W (100 μM). Data are expressed as mean fold-increase (± SEM) over control cells and is representative of three independent experiments. Significance (*p* < 0.05) is indicated by: * vs. control; ** vs. cytomix.

We first examined the effects of cytomix on LA-4 cell NO production. We previously demonstrated that this cytomix treatment regimen resulted in a non-injurious inflammatory microenvironment [25]. Cytomix treatment consisted of supplementing the maintenance medium of confluent LA-4 cells with 0.2 ng/mL each of TNFα + IL-1β + IFNγ for 24 h. Data show that by 24 h, iNOS mRNA was significantly upregulated (>100-fold) with corresponding increases of intracellular iNOS protein relative to control cells (Figure 2A). Furthermore, significant increases in fluorescence of the NO-specific fluorescence probe, DAF-FM diacetate, were detected; while no increase occurred in cells co-treated with 1400W, an iNOS specific inhibitor (Figure 2B). Together, data indicate that cytomix treatment acutely increased NO production though activation of epithelial cell iNOS.

### ROS production and cell injury in cytomix-treated LA-4 cells exposed to DEP

**Figure 3:**
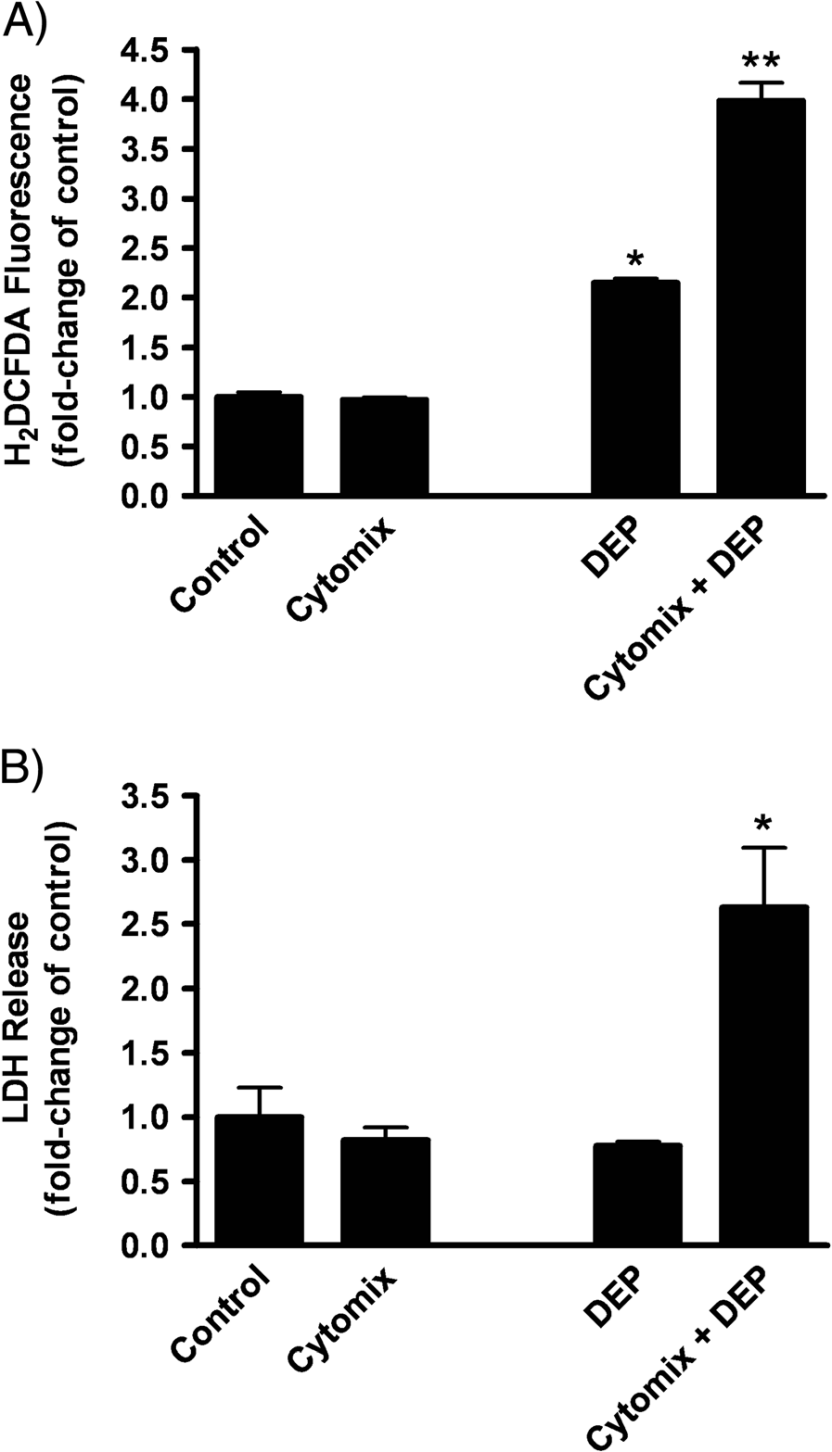
**ROS changes and cell injury in cytomix + DEP-treated cells.** (**A**) ROS production, measured by fluorescence of H_2_DCFDA in saline- and cytomix-treated LA-4 cells exposed to DEP (25 μg/cm^2^ × 2 h). Data are expressed as mean fold increase (± SEM) over control cells and is representative of three independent experiments. Significance ( *p* < 0.05) indicated by: * vs. control; ** vs. DEP. (**B**) Cytotoxicity in saline- or cytomix-treated LA-4 cells with or without exposure to DEP (25 μg/cm^2^ × 24 h) based on LDH release. Data are expressed as mean fold-increase (± SEM) over saline-exposed cells and is representative of three independent experiments. Significantly ( *p* < 0.05) greater injury is indicated by: * vs. All other treatments.

To assess whether DEP exposure (25 μg/cm^2^) differentially impacted the redox status of cytomix-treated cells, we evaluated intracellular ROS production, cytotoxicity, and alterations of reduced (GSH) vs. disulfide (GSSG) forms of the ubiquitous antioxidant, glutathione. Data revealed that cytomix-only treatment did not alter the level of intracellular ROS at 2 h (as detected by changes in H_2_DCFDA fluorescence) (Figure 3A); nor did it cause detectable cytotoxicity by 24 h (based on % LDH leakage) (Figure 3B).

DEP exposure of saline-treated cells elicited increased ROS production by 2 h (Figure 3A); however, exposure was not associated with significant cytotoxicity at 24 h (Figure 2B). Based on increases in intracellular GSH content (30%) and GSH:GSSG molar ratios (17%) at 24 h post-exposure (Table 1), it appeared that “"healthy”" epithelial cells were able to mount an effective antioxidant response during DEP exposure.

**Table 1:** Cellular glutathione 24 h after saline- or cytokine-treated LA-4 cells, with and without DEP

**Cell Groups *n =*** **3/group**	**GSH**	**GSSG**	**GSH:GSSG ratios**
**Saline**	148 ± 2.9	35.7 ± 4.1	4.1 ± 0.4
**Cytomix**	164 ± 12.0	44.2 ± 3.2	3.7 ± 0.4
**DEP**	193 ± 28.9	40.3 ± 3.8	4.8 ± 0.3
**Cytomix + DEP**	394 ± 20.1*	132 ± 2.7*	3.0 ± 0.2

Data are expressed as mean pmol/μg protein (± SEM). Asterisk (*) indicates significantly different than saline-exposed cells ( *p <* 0.05).

On the other hand, DEP exposure of cytomix-treated cells resulted in a greater (4-fold) increase in intracellular ROS at 2 h (Figure 3A), with evidence of commensurate increases in cell injury by 24 h (Figure 3B). In this scenario, it appeared that, despite a significant (>2-fold) increase in cellular GSH levels, cellular redox status could not be maintained as evidenced by the ~30% decline in the GSH:GSSG ratios (Table 1). Together, data reveal that within an inflammatory microenvironment, DEP exposure was associated not only with greater epithelial ROS production, but also oxidative stress and redox imbalance, culminating in overt cytotoxicity.

### Exposure of LA-4 epithelial cells to DEP results in O_2_˙^-^ production

**Figure 4:**
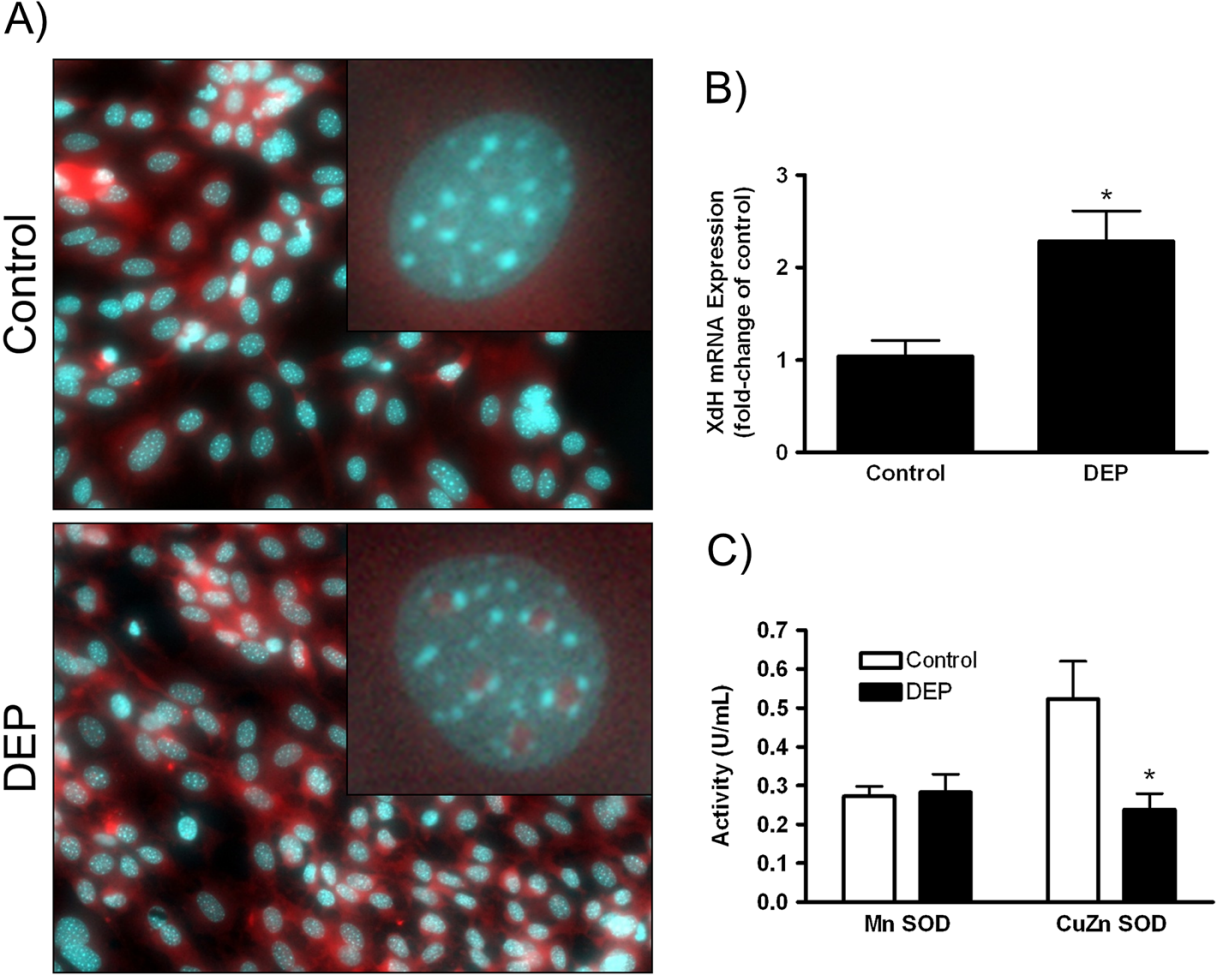
**Superoxide anion production in DEP-exposed cells.** (**A**) DHE fluorescence (red) oxidation by O_2_˙^-^ in DEP-exposed cells (25 μg/cm^2^ × 2 h). DNA was counter-stained with DAPI (blue) and is representative of two independent experiments. (**B**) XdH mRNA expression of saline- and DEP-exposed cells (25 μg/cm^2^ × 24 h). Data are expressed as the mean fold-increase (±SEM) over control cells and is representative of three independent experiments. (**C**) Activity of cytosolic superoxide dismutase (CuZn SOD) and mitochondrial (Mn SOD) SOD from DEP-exposed cells (25 μg/cm^2^ × 24 h). Data are expressed as the mean (± SEM) activity of SOD and is representative of three independent experiments. Significance ( *p* < 0.05) is indicated by: * vs. control.

We next examined more specifically (1) which reactive species were increased and (2) what cellular changes were occurring that may have contributed to the diminished capacity of cells to “"cope”" with the additional oxidative burden. To assess the role of O_2_˙^-^ in DEP-induced responses, LA-4 cells were labeled with the fluorescent probe, DHE. In the presence of O_2_˙^-^, DHE becomes oxidized to ethidium and intercalates with nucleic acids emitting a *red* fluorescence. In contrast to saline-exposed cells which had no nuclear staining and minimal cytoplasmic fluorescence, after 2 h, DEP-exposed cells had increased focal nuclear fluorescence and increased overall fluorescence (Figure 4A) ─ consistent with increased O_2_˙^-^ generation.

We further assessed differential effects on gene expression of xanthine dehydrogenase (XdH), an O_2_˙^-^ generating enzyme. Compared to saline-exposed cells, by 24 h, DEP-exposed cells had a ~2.5-fold increase in XdH expression (Figure 4B). Conversely, activity of the counteracting cytosolic antioxidant factor, superoxide dismutase (CuZn-SOD), was significantly decreased (55%) after DEP exposure. Exposure was without effect on mitochondrial SOD (Mn-SOD) activity (Figure 4C). Together, data indicate that DEP exposure of LA-4 cells resulted in both increased production of O_2_˙^-^ as well as a concomitant decrease in cellular ability to dismute the superoxide anion.

### Role of peroxynitrite in DEP-induced cytotoxicity in cytomix-treated LA-4 cells

**Figure 5:**
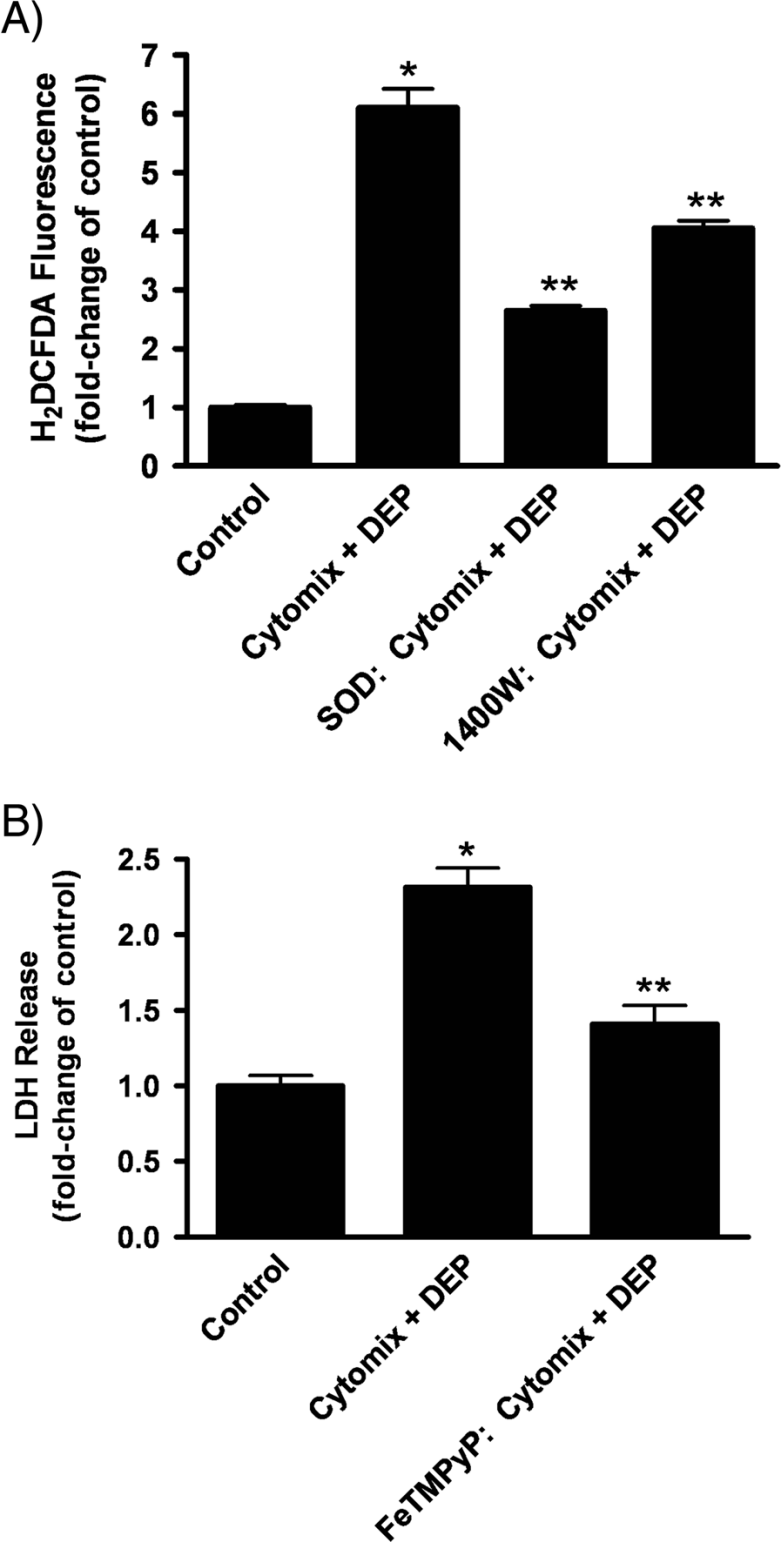
**ROS changes and cell injury in cytomix + DEP-exposed LA-4 cells.** (**A**) ROS production, measured by the fluorescence of H_2_DCFDA oxidation, in cytomix + DEP-exposed (25 μg/cm^2^ × 2 h) LA-4 cells, with or without SOD (200 U/mL) or 1400W (100 μM) treatment. Data are expressed as mean fold-increase (± SEM) over control cells and is representative of three independent experiments. Significance ( *p* < 0.05) indicated by: * vs. control; ** vs. cytomix + DEP. (**B**) Cytotoxicity of cytomix + DEP- exposed cells (25 μg/cm^2^; x 24 h) in the presence of FeTMPyP (10 μM). Data are expressed as mean fold increase (± SEM) of LDH release over control cells and is representative of three independent experiments. Significance ( *p* < 0.05) indicated by: * vs. control; ** vs. cytomix + DEP.

To more specifically implicate involvement of NO vs. O_2_˙^-^ with the increases in H_2_DCFDA fluorescence observed, we next assessed H_2_DCFDA oxidation in the presence of exogenously administered SOD or in cells pretreated with the selective iNOS inhibitor, 1400W. As before, cytomix + DEP-exposed cells had a robust (6-fold) increase in ROS production by 2 h. ROS increases were significantly reduced by supplemental SOD (50% reduction) or 1400W treatment (30% reduction); with neither intervention completely ameliorating the fluorescence increases (Figure 5A).

When both O_2_˙^-^ and NO levels are increased, the two radicals can interact to produce peroxynitrite (ONOO^-^). To elucidate the role of ONOO^-^ in the enhanced cell injury observed during cytomix + DEP exposure, cells were co-treated with FeTMPyP to hasten catalysis of ONOO^-^ (Figure 1A). As before, by 24 h, the cytomix + DEP-exposed cells had significantly greater cell injury (2.3-fold increase) compared to the “"healthy”" control cells; with FeTMPyP treatment significantly decreasing LDH release to near control levels (1.4-fold increase) (Figure 5B). Taken together, data suggest that the increases in NO and O_2_˙^-^ led to generation of ONOO^-^; and that ONOO^-^ production played a significant role in the increased susceptibility of the “"inflamed”" epithelial cells to undergo oxidative/nitrosative stress ─ and hence, cellular necrosis ─ upon exposure to DEP.

### DEP inhalation exposure in cytokine-treated mice

To extend our findings beyond the *in vitro* epithelial model, we similarly administered a mixture of cytokines (TNFα 1.0 ng/g body weight + IL-1β 0.5 ng/g + IFNγ 2.0 ng/g) exogenously into the airways of BALB/c mice to induce *generic* lung inflammation prior to *in vivo* DEP exposure. Initial studies to establish the pulmonary effects of DEP inhalation exposure in healthy mice demonstrated that, 24 h after exposure to 2 mg/m^3^ DEP × 4 h/d × 2 d, mice developed mild lung inflammation (with significant increases in neutrophils and lymphocytes in BAL fluid), but without evidence of lung injury. Based on minor increases in GSH and GSSG levels (20%), DEP-exposed mice had mounted low-level antioxidant lung responses (Table 2).

**Table 2:** BAL fluid indices and lung glutathione in mice 24 h after nose-only air or DEP inhalation exposure

	**Filtered Air**	**DEP**
**BAL fluid cells per lung (x10**^**3**^**)**	*n =* 4	*n =* 4
Total Cells	85.3 ± 7.4	111 ± 12.3
Macrophages	84.9 ± 7.3	108 ± 11.1
Neutrophils	0.1 ± 0.1	1.4 ± 0.40*
Lymphocytes	0.2 ± 0.1	1.7 ± 0.7*
**BAL fluid biochemistries**		
LDH (U/mL)	18.8 ± 3.1	12.6 ± 1.8
Total Protein (μg/mL)	62.9 ± 5.6	49.6 ± 11.1
Albumin (μg/mL)	15.1 ± 0.9	10.9 ± 3.8
**Lung glutathione (nmol/g tissue)**		
GSH	4012 ± 371	4860 ± 561
GSSG	356 ± 35.1	427 ± 39.8
GSH:GSSG ratios	11.7 ± 1.5	11.3 ± 0.4

DEP exposures were at 2 mg/m^3^ for 4 h/d × 2 d. Data are expressed as the mean ± SEM. Asterisk (*) indicates significantly different than air-exposed mice ( *p <* 0.05).

Studies to evaluate effects of cytomix treatment demonstrated that mice developed significant acute lung inflammation (peaking at 48 h) along with transient edema (based on albumin increases in BAL fluid without LDH increase) (Table 3). Although NO changes were not assessed herein, pulmonary edema has been associated with increased NO production in animal models of acute lung injury [29,30]. Of note, both neutrophils and lymphocytes were significantly increased, consistent with MIP-2 and RANTES production, respectively. By 48 h, based on increased GSH (20%) and GSH:GSSG ratios (30%), it appeared that cytokine treatment was also associated with mild antioxidant responses (Table 3).

**Table 3:** BAL fluid indices and lung glutathione in mice 48 h after saline- or cytokine-treatment

	**Saline**	**Cytomix**
	***n =*** **4**	***n* = 4**
**BAL fluid cells per lung (x10**^**3**^**)**		
Total Cells	88.7 ± 4.8	264 ± 25.5*
Macrophages	72.5 ± 9.5	101 ± 7.1
Neutrophils	14.7 ± 6.7	119 ± 13.1*
Lymphocytes	1.4 ± 0.7	57.1 ± 16.7*
**BAL fluid biochemistries**		
LDH (U/mL)	37.5 ± 9.1	55.3 ± 13.7
Total Protein (μg/mL)	67.6 ± 5.2	126.6 ± 21.4
Albumin (μg/mL)	15.2 ± 1.9	24.6 ± 2.5*
**Lung glutathione (nmol/g tissue)**		
GSH	4050 ± 484	4908 ± 1080
GSSG	331 ± 12.5	305 ± 74.1
GSH:GSSG ratios	12.3 ± 1.6	16.1 ± 3.5

Data are expressed as means (± SEM). Asterisk (*) indicates significantly different than saline-exposed mice ( *p <* 0.05).

**Figure 6:**
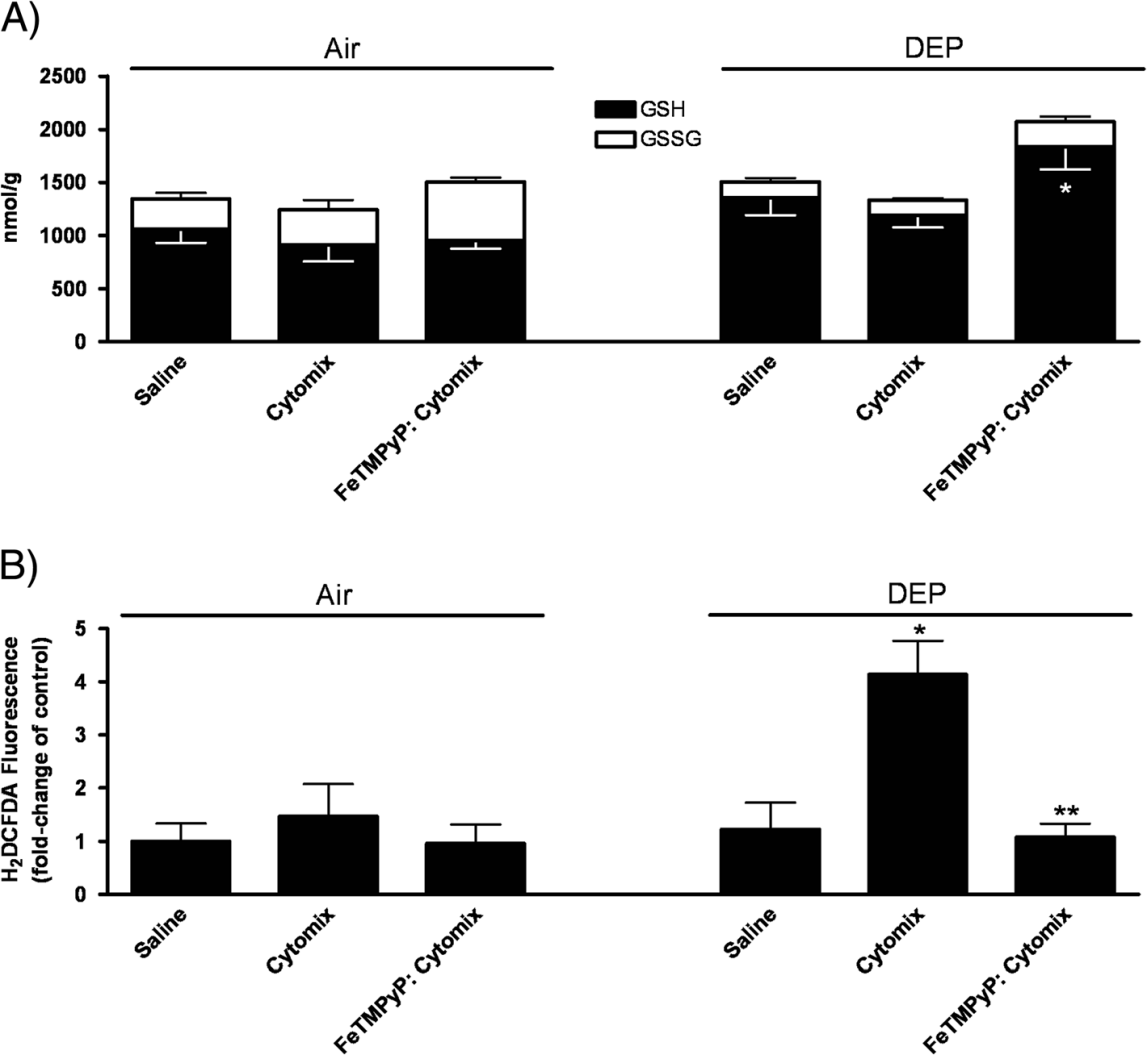
**Day 4 comparison of mice.** (**A**) lung glutathione levels and (**B**) ROS production in cells obtained by lung lavage in saline- or cytomix-treated mice, 24 h after exposure to air or DEP for 2 days (mean ± SEM; *n =* 4/group). Data are expressed as the mean nmol/g of lung tissue (± SEM) for GSH or GSSG. Significance ( *p* < 0.05) indicated by: * vs. DEP-cytomix. For ROS cell production, data are expressed as mean fold increase (± SEM) over saline + air-exposed mice. Significance ( *p* < 0.05) indicated by: * vs. air, cytomix, DEP; ** vs. cytomix + DEP.

In the main study, subsets of mice were treated with phosphate-buffered saline (PBS) or cytomix as above (Day 0), and 48 h later, mice underwent nose-only inhalation exposure to filtered air or the same DEP particles as above (Day 2 and 3) (Figure 1B). By Day 4, no significant differences in body weights were observed across the treatment groups. Analysis of BAL fluid on Day 4 failed to reveal significant changes in cellular or biochemical indices, or in lung glutathione levels, across the treatment groups (Table 4; Figure 6A). Somewhat unexpectedly, unlike the earlier study, DEP-only exposed mice did not develop detectable lung inflammation. Furthermore, it appeared that the inflammatory response induced by the single cytomix treatment had largely resolved by Day 4. However, we again noted that both saline + DEP- and cytomix + DEP-exposed mice had mildly increased GSH (~30%) levels along with significantly increased GSH:GSSG ratios (2.5-2.9-fold), compared to corresponding subsets of air-exposed mice. Results are indicative that, as above, DEP exposure was associated with moderate antioxidant lung responses (Table 4; Figure 6A).

**Table 4:** BAL fluid indices and lung glutathione ratios in saline- or cytomix-treated mice, 24 h after exposure to air or DEP for 2 consecutive days

	**Air**			**DEP**		
***N =* 4/group**	**Saline**	**Cytomix**	**FeTMPyP: Cytomix**	**Saline**	**Cytomix**	**FeTMPyP: Cytomix**
**BAL fluid cells per lung (x10**^**3**^**)**						
Total Cells	110 ± 52	96.4 ± 22	109 ± 11	84.8 ± 21	107 ± 35	84.3 ± 10
Macrophages	101 ± 44	89.3 ± 20	101 ± 11	83.2 ± 21	96.4 ± 33	78.0 ± 9.1
Neutrophils	0.6 ± 0.10	4.1 ± 1.8	5.5 ± 1.5	0.4 ± 0.1	7.5 ± 2.6	4.7 ± 0.8
Lymphocytes	0.9 ± 0.6	3.0 ± 0.7	3.0 ± 1.2	1.2 ± 0.5	2.8 ± 0.7	1.6 ± 0.7
**BAL fluid Biochemistries**						
LDH (U/mL)	37.0 ± 9.8	38.8 ± 8.6	38.1 ± 1.6	23.5 ± 1.3	43.7 ± 6.7	36.4 ± 2.6
Total Protein (μg/mL)	66.8 ± 17.8	82.0 ± 8.4	74.2 ± 1.7	68.1 ± 2.4	88.1 ± 16.9	75.9 ± 2.3
Albumin (μg/mL)	15.9 ± 3.1	16.6 ± 0.5	14.1 ± 0.8	16.9 ± 0.4	14.7 ± 1.7	14.7 ± 0.6
**Lung GSH:GSSG**	3.9 ± 0.4	3.1 ± 0.6	1.7 ± 0.1	9.9 ± 1.2*	9.2 ± 1.8*	8.8 ± 1.4*

Data are expressed as the mean (± SEM) in saline- or cytomix-treated mice, 24 h after exposure to air or DEP for 2 consecutive days (2 mg/m^3^; 4 h/d × 2 d). Asterisk (*) indicates significantly different than saline-exposed mice ( *p <* 0.05).

Despite the negligible effects on lung injury and inflammation, results revealed that cytomix + DEP-exposed mice had significantly increased ROS production (4-fold) in phagocytic cells obtained via lung lavage (based on H_2_DCFDA fluorescence) (Figure 6B). Fluorescence increases were inhibited, to control levels, in mice receiving FeTMPyP treatment (Day −1 to 4) (Figure 6B). We further observed that in these mice, lung tissue GSH levels were increased (~55%) over that of the cytomix + DEP-exposed mice that did not receive FeTMPyP systemically (Figure 6A).

Collectively, data indicate that *in vivo* exposure to this DEP regimen was associated with low-level pulmonary oxidative stress, to which, both saline- and cytomix-treated mice were able to mount effective antioxidant responses, thereby preventing significant lung injury or inflammation. Nevertheless, in mice with pre-existing lung inflammation, DEP exposure was associated with significantly greater ROS production within lung phagocytes. Because H_2_DCFDA fluorescence was attenuated by FeTMPyP treatment, increases were related to ONOO^-^. We further speculate that because the FeTMPyP-treated mice did not have to “"cope”" with the additional phagocyte ONOO^-^production, there was an *overshoot* in the overall lung antioxidant response, as evidenced by significant (~2-fold) increases in lung GSH levels 24 h after DEP-exposure, relative to saline + air-exposed mice (Figure 6A).

## Discussion

Maintaining redox balance in the lung is a dynamic process. It is especially challenging within the air passageways and alveolar spaces, where surface epithelial cells and resident phagocytes are exposed to ─ and provide the first line of defense against ─ a wide range of inhaled biologic (e.g., bacteria, viruses, allergens) and environmental agents (e.g., ozone, PM). In the present investigation, we used relatively simplistic *in vitro* and *in vivo* murine models of cytokine-induced epithelial and lung inflammation, respectively, to demonstrate the potential for NO (increased during inflammatory conditions) and ROS (increased as a consequence of traffic PM exposure) to “"co-operate”" to produce reactive oxidative as well as reactive nitrosative species (RNS) within PM-exposed lung cells. Specifically, we show that epithelial cells exposed to OC-rich DEP within an inflammatory microenvironment incur greater ROS/RNS burden and corresponding epithelial cytotoxicity; and that cytomix + DEP-exposed mice incur greater ROS/RNS production in lung phagocytes.

What evidence exists that respiratory inflammation is associated with increased NO production in people? Asthma, COPD, bronchitis, and rhinitis all represent a spectrum of respiratory disorders in which the clinical manifestations are orchestrated by, or in large part the result of, underlying inflammatory processes. Whereas all three nitric oxide synthase isoforms are present in the respiratory tract, clinical studies show that exhaled NO (eNO) levels are higher in patients with asthma [31,32], COPD [33] and seasonal rhinitis [34]. The magnitude of eNO increase is often proportionate to the degree of symptomatology, inflammation, and aeroallergen sensitization. Elevations are, in large part, derived from iNOS localized within the inflamed epithelium [35,36] ─ with highest iNOS expression found in epithelial cells of patients with severe asthma [37]. Additional studies reveal eNO increases in association with childhood exposure to traffic emissions [38], ambient air pollution [39], or early life exposure to PAHs [40]. Moreover, recent studies suggest that such exposures may influence genetic and epigenetic variations in the iNOS promoters [41,42].

How could these changes contribute to the adverse respiratory outcomes associated with traffic emissions in humans? Lung epithelial dysfunction is considered central to development of asthma; with insults such as air pollutants serving not simply as triggers for disease exacerbation, but also as playing critical roles in the origin and progression of airway and lung pathology [43]. A growing body of literature further implicates impaired antioxidant defenses and disturbances in oxidation/reduction (redox) balance as risk factors for asthma development and asthma severity [44,45]. Accumulating *in vitro* and *in vivo* experimental studies have shown that traffic PM exposure is associated with increased lung oxidant burden related to increased ROS such as O_2_˙^-^[46-49]. Other studies of DEP-exposed rodents reveal concomitant increases in NO and ONOO^-^ in BAL fluid cells [50,51].

In the present investigation, data demonstrated that despite ROS increases in DEP-exposed epithelial cells, significant cytotoxicity was *not* observed unless cells had been exposed within an inflammatory microenvironment ─ suggesting a cooperative role of particle-induced ROS with existing lung inflammatory mediators. We clearly show how DEP effects were mediated by: (1) increased ROS (including O_2_˙^-^) production related to increased XdH expression and reduced CuZn SOD activity; and (2) increased RNS production owing to interaction of O_2_˙^-^ with cytokine-induced, NO, to generate peroxynitrite. We confirm that DEP-induced epithelial effects could be partially ameliorated by providing additional SOD or blocking iNOS induction. Epithelial cell and *in vivo* phagocyte effects were, by and large, *prevented* by accelerating catalysis of the longer-lived, peroxynitrite radical, through administration of the iron porphyrin, FeTMPyP.

Normally, in health, respiratory tract epithelial cells and lung phagocytes work in concert to provide protection against inhaled microorganisms. The ability to greatly increase production of NO (and related RNS) is key to respiratory system innate immunity. In neutrophils, for example, cooperative action of NO and O_2_˙^-^ imparts their ability to kill ingested microbial pathogens [52]. Likewise, after microbial phagocytosis by alveolar macrophages, iNOS activation and respiratory “"burst”" activity similarly mediate pathogen clearance [53]. A variation on this theme occurs in airway epithelial cells during whooping cough (pertussis) infection as infected epithelial cells respond to IL-1 by increasing iNOS. Excess NO induces epithelial autotoxicity and shedding of infected cells, thereby limiting spread of pertussis organisms to adjacent healthy cells [54]. Consequently, the lung has developed an extensive capacity to withstand oxidative and nitrosative insult, at least on a short-term basis, as required during acute inflammatory response to infectious agents.

Respiratory pathogens, on the other hand, have evolved intricate NO-sensing capabilities and defense mechanisms of their own [55]. Murine models of viral infection reveal major shifts in the cellular and temporal distribution of lung antioxidant enzymes during, for example, influenza pneumonia [56]. RSV infection can similarly induce significant down-regulation of host airway antioxidant processes (e.g., SOD activity), which in infants (possibility owing to immature antioxidant defense mechanisms), can result in extensive oxidative epithelial damage and severe bronchiolitis [57].

Air pollution PM has inherent oxidant properties that are highly correlated with OC (e.g., PAH) and metal content [58]. While such characteristics no doubt contribute to the overall oxidant effects of traffic PM, it is likely that ─ owing to their resemblance to inhaled microorganisms ─ *in vivo* toxicity and health effects are mediated largely by generation of ROS/RNS within exposed cells during futile attempts by innate host defenses to respond to inhaled fine PM as if they were potential pathogens. As such, cellular redox modulation, whether related to infection or PM exposure, appears to be deeply entangled with host inflammatory responses. We focused, therefore, on DEP-induced changes in glutathione because it is the most abundant intracellular antioxidant thiol, and is central to redox defense during oxidative/nitrosative stress [59]. The diverse functions of GSH (γ-glu-cys-gly) originate from the sulfhydryl group in cysteine, enabling GSH to participate in redox cycling. Glutathione redox changes regulate not only signal transduction and airway inflammation, but also airway reactivity and hyperresponsiveness [60].

However, if and when simultaneous production of NO and O_2_˙^-^ occur, excess RNS levels can exceed available cellular, and even tissue antioxidant capacity. Excessive RNS ─ not unlike excess ROS ─ can induce DNA damage, modify lipids [27,28,61], and cause protein misfolding and dysfunction [62]. In response to misfolded protein, the *unfolded protein response* (UPR) triggers a series of intracellular events aimed at either eliminating rogue (damaged) cells by inducing apoptosis [62] or allowing cells to overcome the consequences of the stress by altering expression of anti-oxidant response genes, cell cycle progression, or inflammatory cascades [63]. Our data similarly showed that significant epithelial damage occurred *only* if DEP exposure was associated with decreased GSH:GSSG ratios. This occurred *only* in the DEP-exposed “"inflamed”" cells *in vitro*. It appeared that as cumulative oxidative/nitrosative stress exceeded LA-4 cell capacity to maintain adequate redox status (i.e., GSH:GSSG ratios decreased), epithelial function became progressively impaired, resulting in cellular apoptosis and/or necrosis.

There were several limitations to this investigation. First, alveolar epithelial cell responses to PM may differ from that of airway epithelial cells, and further, particle effects in cell lines may or may not reflect that of their corresponding primary cells of origin. To this end, we previously showed that both LA-4 cells and primary murine airway epithelial cultures (established at an air-liquid interface) were more susceptible to DEP-induced effects when exposed within this cytokine-induced inflammatory microenvironment [25]. Henceforth, we used the LA-4 cells as general surrogates to assess particle effects on surface cells of the respiratory tract. These generic *in vitro* and *in vivo* inflammatory models may also fail to recapitulate key features of disease processes occurring in asthmatics (i.e., T_H_2- or eosinophil-mediated effects). However, if traffic PM exposure was to only influence allergen-specific processes, effects would be unlikely to explain the health associations noted in patients with COPD, chronic bronchitis, or 50% of adult asthmatics who are not overtly atopic [64]. It was because augmented health effects are associated with a broad range of inflammatory conditions, that we intentionally developed this cytokine combination to model a *generic* inflammatory state. Nonetheless, efforts are in progress to refine the *in vivo* cytokine protocol to better simulate longer-lived, lower-level, inflammation in the mice.

Although expression of XdH was increased during DEP exposure, XdH (normally involved in the metabolism of purines), can also be converted to xanthine oxidase, thus contributing to still greater O_2_˙^-^ production. DEP influences on the mitochondrial respiratory chain or other enzyme systems (e.g., NADPH oxidases, P_450_ enzymes) cannot be ruled out as important sources of O_2_˙^-^ production [65]. Likewise, other oxidant (e.g., H_2_O_2_, ^∙^OH) or nitrosative [e.g., nitrosonium (NO^+^), nitroxyl (NO^-^), HNO] species may have contributed to DEP-induced redox changes.

Herein, relatively high-dose, short-term exposures to DEP were utilized. In so doing, this study was primarily designed to examine differential effects of DEP in healthy vs. diseased states, and as such, showed that under inflammatory conditions, LA-4 cells were 10-fold more susceptible to DEP-induced epithelial damage [25]. Although *real world* exposures to urban air pollution are lower-level, they are also chronic and involve multiple pollutants. It may be relevant that school buses contribute substantially to DEP exposure in children, with onboard PM_2.5_ levels being four-fold higher than ambient levels, and two-fold higher than roadway levels [66]. Aerosol particle counts at schools are 2.3- to 4.7-fold higher than areas without bus-related traffic [67]. Likewise, in urban schools serviced by diesel buses, ambient near- and in-school PM_2.5_ fluctuations correspond temporally with bus drop-off hours and are highest in schools with the greatest number of buses in operation [68].

We further acknowledge that DEP composition can vary, and hence we used select DEP samples that resembled tunnel traffic PM (in terms of relative EC and OC content) to represent traffic-based PM. Other components of near-road emissions, for example, gaseous and semivolatile compounds [69] and metals from tire, brake, and rotor wear [70] reportedly have pro-oxidant properties and also participate redox cycling. It is possible, therefore, that similar to these diesel-derived particles, under inflammatory conditions, exposure to other traffic-related oxidants may similarly enhance ROS/RNS production.

Results of the present investigation provide biologic plausibility for the ever increasing epidemiological database associating traffic exposure with adverse health impact, especially in individuals with pre-existing respiratory diseases [1,8,71]. Relatedly, in asthmatics, airway redox balance appears to be shifted toward a more oxidized state [45] and the epithelial barrier is already somewhat compromised [72]. In poorly controlled asthma, alveolar macrophages are prone to apoptosis and phagocytosis is impaired [73]. Experimental studies implicate glutathione depletion and redox imbalance in these phagocyte deficits [74]. Our data similarly implicates cellular redox imbalance as a precursor to both: (1) phagocyte and epithelial cell signaling and associated inflammatory processes and (2) epithelial injury and barrier dysfunction. These results support novel therapeutic approaches designed to increase the airways’ resistance against the inhaled environment agents rather than focusing solely on suppression of inflammation [43]. Ancillary medical and nutritional interventions may be warranted, particularly in children and at-risk populations [75-77].

Based on the above experimental and clinical findings, we put forth the supposition that in at-risk populations, traffic-associated ROS/RNS production further compromises epithelial barrier and phagocytic cell function, thereby allowing penetration of inhaled pathogens and allergens deeper into lung tissue. In so doing, chronic traffic exposure would predispose individuals to repeated respiratory infection and immune cell antigen exposure, respectively; which overtime would promote and elicit end organ expression of atopic asthma [72]. This scenario is supported by epidemiologic reports worldwide associating early life or childhood exposures to air pollution from traffic with development of respiratory infections and asthmatic and allergic symptoms [2-4,6,15,20,22,78].

## Conclusions

Under inflammatory respiratory conditions, adverse health effects related to exposure to traffic emissions appear to involve a complex interplay between radical-generating capacities of traffic particles with *in vivo* mediators related to ongoing inflammatory processes. In highly exposed cells (i.e., surface epithelial cells and phagocytic cell populations), repeated exposure to traffic emissions may result in dual ROS + RNS insult which, in at-risk populations, exceeds cellular capacity to maintain redox balance. In so doing, exposure could cause and perpetuate epithelial barrier dysfunction [72] and alter innate and adaptive lung immune response [73]. Our results provide a possible unifying mechanism to explain why individuals with a variety of pre-existing inflammatory diseases are particularly susceptible to developing adverse respiratory health effects during acute and chronic exposure to traffic emissions.

## Materials and methods

### DEP samples

For the *in vitro* studies, particles generated in 1999 by a diesel powered automobile were used (a gift from Dr. Daniel Costa, US EPA). As previously reported, these particles closely resembled urban tunnel traffic emissions in that they were comprised of 35% OC, 35% EC, and low levels of soluble metal [25]. Due to limited quantities of this sample, the murine inhalation studies were performed using a DEP sample of comparable OC content (~35%) that had been generated in bulk and characterized by the Inhalation Toxicology Facility (US EPA, Research Triangle Park, NC) as described previously [79].

### Cell culture treatment and DEP exposure

LA-4 cells, a murine alveolar type II-like epithelial cell line (ATCC, Manassas, VA; passages 49–55) were grown to confluence for 1–2 days in Ham's F12K medium with 10% FBS. Upon nearing confluence, cells were maintained in serum-free Ham's F12K medium with select growth factors and 0.5 mg/mL BSA (Sigma, St. Louis, MO) as described previously [25]. Cytomix treatment consisted of supplementing the maintenance medium with 0.2 ng/mL each of TNFα + IL-1β + IFNγ (R&D Systems, Minneapolis, MN) for 24 h. Fresh medium (without cytomix) was then applied and cells were exposed to DEP at 25 μg/cm^2^ for 2 h (for fluorescent end points) or 24 h (for cytotoxicity) (Figure 1A). For DEP exposures, freshly prepared particle suspensions in saline (sonicated 3-times on ice; 10 sec each) were spiked into medium. In select experiments, to block iNOS expression, cells were pre-treated with the selective iNOS inhibitor, 1400W dihydrochloride (100 μM; Sigma, St. Louis, MO) for 24 h prior to applying cytomix. To decrease O_2_˙^-^ levels, superoxide dismutase (SOD; 200 U/mL; Sigma, St. Louis, MO) was added to the medium 1 h prior to DEP exposure. To catalyze decomposition of peroxynitrite, cells were treated with a synthetic porphyrin complexed to iron (FeTMPyP; 10 μM; Cayman Chemical, Ann Arbor, MI) during DEP exposure. Unless otherwise indicated, *n =* 4 wells per treatment group per time point assessed.

### *In vitro* assessments

#### Nitric oxide

Changes in iNOS gene expression, protein, and NO production were assessed in LA-4 cells 24 h after addition of cytomix. Total RNA was isolated using an RNeasy kit (Qiagen, Valencia, CA). cDNA synthesis and realtime PCR using gene-specific primers and probes for iNOS, xanthine dehydrogenase (XdH), and β-actin (Applied Biosystems, Foster City, CA) were performed using SuperScript III Platinum One-Step Quantitative RT-PCR System (Invitrogen, Carlsbad, CA). Differential expression was determined using the 2^-∆∆CT^ method [80]. Cell lysate iNOS protein levels were assessed by separating equal amounts of protein on E-PAGE 8% gels (Invitrogen, Carlsbad, CA). Protein was transferred, blocked for 1 h, probed overnight at 4°C with antibodies to iNOS (1:500; BD Transduction Laboratories, San Jose, CA) or β-actin (1:5000; Sigma, St. Louis, MO), washed again, and incubated for 1 h with corresponding secondary antibodies. Signals were detected using chemiluminescence (LumiGlo, Cell Signaling Technology, Danvers, MA) with images acquired using an Alpha Innotech 8900 imaging station (San Leandro, CA). NO production was assessed after 30 min incubation with the fluorescent probe, DAF-FM (10 μM; Invitrogen). Fluorescence was quantified on a plate reader (Packard FluoroCount BF10000).

#### Cytotoxicity

Using a commercially available kit for lactate dehydrogenase (LDH) (Thermo Fisher Diagnostics, Middletown, VA), LDH % release was used to assess LA-4 cytotoxicity. Cell lysate protein was determined using a kit (Thermo Scientific, Rockford, IL). Assays were modified and adapted for use on the KONELAB Arena 30 clinical chemistry analyzer (Thermo Clinical Labsystems, Espoo, Finland).

#### Intracellular ROS production

Changes in generic ROS production were evaluated by incubating LA-4 cells for 30 min with the non-specific fluorescent probe, 2',7'-dichloro-fluorescein diacetate (H_2_DCFDA; 10 μM; Invitrogen, Carlsbad, CA) followed by exposure to DEP for 2 h. Fluorescence was quantified using a fluorescence plate reader. Changes in intracellular O_2_˙^-^ levels were detected in LA-4 cells grown on chamber slides and incubated for 30 min with the probe, dihydroethidium (DHE; 10 μM; Invitrogen, Carlsbad, CA) and then exposed to DEP for 2 h. Cells were imaged using a fluorescent microscope (Nikon Eclipse Ti; Nikon Elements software; Nikon Instruments, Inc.).

#### SOD Activity

After probe sonication, cell lysates were placed in cold 20 mM HEPES buffering solution, centrifuged, and supernatants assayed for SOD activity as per the manufacturer's instructions (RANSOD, RANDOX Laboratories Ltd, Co., Antrim, UK).

#### Glutathione

As described previously [25], dislodged LA-4 cells were treated with cold 10% perchloric acid containing 0.4 M boric acid (Sigma, St. Louis, MO). After centrifugation (20 min, 4°C, 20,000 g), cell-free supernatants were treated with dansyl chloride (Sigma) to label the reduced (GSH) and disulfide (GSSG) glutathione fractions. After gradient HPLC separation (Discovery C_18_ columns; Sigma), the fluorescent products of dansylated GSH and GSSG, as well as GSH and GSSG standards, were acquired (excitation at 335 nm; emission at 515 nm) using a fluorescence detector (Model 1100; Agilent Technologies, Santa Clara, CA) and quantified (ChomPerfect Chomatography Data System software; Justice Laboratory Software, Denville, NJ).

### DEP inhalation exposures

#### Mice

Female BALB/c mice (Charles River Labs, Wilmington, MA), 12–16 weeks of age (19–23 g) were housed in an AAALAC–accredited facility maintained on a 12 h light/dark cycle. Food and water were provided *ad libitum* except during the DEP exposures. Mice were acclimated to the facilities and nose-only exposure tubes prior to use. All procedures were approved by the Institutional Animal Care and Use Committee.

#### Cytomix treatment

Mice were briefly anesthetized with vaporized isoflurane (Webster Veterinary Supply Inc., Sterling, MA) to administer 50 μL of either sterile PBS or cytomix into the lungs via oropharyngeal aspiration. Based on dose-range finding studies with these cytokines individually, and in combination (data not shown), the cytomix regimen used herein consisted of a single treatment with TNFα (1.0 ng/g of body weight) + IL-1β (0.5 ng/g) + IFNγ (2.0 ng/g) R&D Systems, Minneapolis, MN.

#### DEP exposures

Using the EPA string-generation particle exposure system, mice were placed in separate 24-port nose-only flow-by inhalation chambers and exposed to filtered air or resuspended DEP [81]. Particle concentration and size distribution were monitored and confirmed as previously described [82]. A pilot inhalation study in healthy mice was performed to determine a DEP exposure regimen that would induce mild, but detectable, lung inflammation. In the formal DEP inhalation study (Figure 1B), mice were pre-treated (Day 0) with phosphate-buffered saline (PBS) or cytomix as above, and 48 h later underwent nose-only inhalation exposure to filtered air or DEP (2.0 mg/m^3^) for two consecutive days (4 h/d × 2 d) (Day 2 and 3). Mice were euthanized (Day 4) via anesthetic overdose (Euthasol, 150–200 mg/kg, i.p.) followed by exsanguination. In a subset of mice, FeTMPyP was administered (10 mg/kg, i.p.) 24 h prior to cytokine treatment and daily until euthanasia (Day −1 to 4).

### *In vivo* assessments

Mice were observed daily. Immediately following euthanasia, mice were weighed, tracheas cannulated and the left lung lobes ligated, resected, and snap frozen (−80°C). The remaining accessory and right lung lobes were lavaged with three separate volumes (0.6 mL) of HBSS. Pooled BAL fluid was centrifuged (800 g × 10 min). Resulting supernatants were analyzed using commercially available kits for total protein (Thermo Scientific, Rockford, IL), albumin (DiaSorin, Stillwater, MN), and LDH (Thermo Fisher Diagnostics, Middletown, VA) adapted for the KONELAB Arena 30 analyzer. Pelleted cells were re-suspended in HBSS and cells enumerated using a Z1 Coulter counter (Coulter, Hialeah, FL). Differential cell counts (200 cells/slide) were performed on cytospin (Shandon Pittsburgh, PA) preparations stained with a modified Wright-Giemsa stain on an automated slide stainer (Hematek 2000, Miles Inc., Elkhart, IN). Data are expressed as the total number of cells retrieved during the lavage procedure. Intracellular ROS production was assessed in 10,000 cells that were plated onto cell culture plates (in HBSS), labeled with the H_2_DCFDA probe (10 μM) for 30 min, quantifying fluorescence as above.

Lung GSH and GSSG levels were determined by homogenizing the frozen lung tissue in cold 4% perchloric acid PCA containing 0.2 M boric acid, 4 mM diethylenetriaminepentaacetic acid. Homogenates were centrifuged and supernatants labeled with dansyl chloride, separated with HPLC, quantified as above, and normalized to tissue mass.

### Statistical analysis

Expressed as the mean ± SEM, data were analyzed using an analysis of variance (ANOVA) and where relevant, Bonferroni post-hoc testing for comparisons between multiple groups (GraphPad Prism 4.0.2). A value *p* < 0.05 was considered to reflect statistically significant effects.

## Abbreviations

BAL: Bronchoalveolar lavage; DEP: Diesel exhaust particles; DHE: Dihydroethidium; EC: Elemental carbon; eNO: exhaled NO; GSH: Reduced glutathione; GSSG: Glutathione disulfide; HBSS: Hank's balanced salt solution; H_2_DCFDA: 2',7'-Dichloro-fluorescein diacetate; IFNγ: Interferon gamma; IL-1β: Interleukin-1 beta; iNOS: inducible nitric oxide synthase; i.p.: intraperitoneal; LDH: Lactate dehydrogenase; NO: Nitric oxide; OC: Organic carbon; ONOO^-^: peroxynitrite; PAH: Polycyclic aromatic hydrocarbons; O_2_˙^-^: Superoxide anion; PBS: Phosphate buffered saline; RNS: Reactive nitrogen species; ROS: reactive oxygen species; SEM: Standard error of the mean; SOD: Superoxide dismutase; TNFα: Tumor necrosis factor alpha; XdH: Xanthine dehydrogenase.

## Competing interests

The authors declare that they have no competing interests.

## Authors’ contributions

NDM participated in the design, co-ordination, and execution of all phases of the *in vitro* and *in vivo* studies and also performed molecular assessments, statistical analysis, data interpretation, and drafting the manuscript. AJL participated in the *in vivo* studies, performed the BAL cell fluorescence assays, and helped to draft the manuscript. RS participated in the *in vivo* studies, performed the HPLC procedures and analyses, and helped to draft the manuscript. ADL designed and performed the *in vivo* inhalation exposures. JHR performed all biochemical analyses and assisted with data interpretation. JAD participated in the study concept and design, *in vivo* procedures, data interpretation, and drafting of the manuscript. All authors have read and approved this manuscript.

## Disclaimer

This paper has been reviewed by the National Health and Environmental Effects Research Laboratory, US EPA, and approved for publication. Approval does not signify that the contents necessarily reflect the views and policies of the agency, nor does the mention of trade names or commercial products constitute endorsement or recommendation for use.
